# Neonatal Food Protein-Induced Enterocolitis: Current Insights and Knowledge Gaps

**DOI:** 10.3390/jcm14072461

**Published:** 2025-04-03

**Authors:** Enza D’Auria, Cristina Ferrigno, Stefano Pellicani, Anna Di Gallo, Gian Vincenzo Zuccotti, Massimo Agosti, Maria Elisabetta Baldassarre, Silvia Salvatore

**Affiliations:** 1 Allergy Unit-Department of Pediatrics, Buzzi Children’s Hospital, 20154 Milan, Italy; cristina.ferrigno@unimi.it (C.F.); anna.digallo@unimi.it (A.D.G.); 2 Department of Biomedical and Clinical Sciences, University of Milan, 20157 Milan, Italy; gianvincenzo.zuccotti@unimi.it; 3 Department of Biomedical Science and Human Oncology, Aldo Moro University of Bari, 70124 Bari, Italy; stefano.pellicani@gmail.com; 4 Department of Pediatrics, Buzzi Children’s Hospital, 20154 Milan, Italy; mariaelisabetta.baldassarre@uniba.it; 5 Department of Medicine and Technical Innovation, Pediatrics, Hospital “F. Del Ponte”, University of Insubria, 21100 Varese, Italy; massimo.agosti@uninsubria.it (M.A.); silvia.salvatore@uninsubria.it (S.S.)

**Keywords:** FPIES, allergic enterocolitis, neonatal allergy, vomiting, food allergy, elimination diet, Food Protein-Induced Enterocolitis Syndrome

## Abstract

Acute and chronic Food Protein-Induced Enterocolitis Syndrome (FPIES) has been well characterized in children; otherwise, neonatal FPIES (N-FPIES) remains poorly understood. In terms of pathophysiology, neonatal FPIES appears to have a more prevalent TH2 response and is characterized by specific clinical features that make the diagnosis challenging. Genetic and environmental risk factors may predispose to the development of FPIES. Recent evidence indicates that a characteristic microbiota signature may lead to barrier dysfunction, reduced regulatory T cells, and abnormal intestinal production of serotonin, responsible for the symptoms of FPIES. Regarding clinical presentation, newborns with FPIES may not fully meet the current guideline’s diagnostic criteria at disease onset, being more similar to clinical entity specific of neonatal age than to acute FPIES in infants and children. Hence, differentiation from other neonatal medical and surgical conditions—particularly necrotizing enterocolitis (NEC)—remains a critical challenge for clinicians. This present review highlights our current understanding of N-FPIES, in term of pathophysiology, clinical presentation diagnosis, and treatment strategies. Refining diagnostic criteria for N-FPIES represents a clinical priority to help physicians in diagnosing and managing this challenging condition. Last, but not least, larger clinical trials are needed to optimize treatment practices in term and preterm newborns with FPIES.

## 1. Introduction

Initial descriptions of Food Protein-Induced Enterocolitis Syndrome (FPIES) depicted infants under nine months old with severe vomiting and diarrhea in response to cow’s milk (CM) or soy-based formula [1,2]. Over the years, different definitions of FPIES have been adopted with a broader range of foods implicated in triggering this allergic reaction [2]. A major advancement in knowledge came in 2017 with the establishment of specific diagnostic criteria [3]. The Consensus International Guidelines firstly define two phenotypes, acute and chronic FPIES. Acute FPIES is the most common type of FPIES. According to the guidelines, patients must have one major criterion and at least three minor criteria.

The major criterion is vomiting occurring 1–4 h postingestion of a suspected food, without accompanying IgE-mediated allergic skin or respiratory symptoms. Nine minor criteria were also defined, including a second episode of repetitive vomiting upon re-exposure to the same food, vomiting 1–4 h after ingestion of a different food, lethargy, marked pallor, admission to the emergency department, intravenous fluid requirement, diarrhea within 24 h (often occurring 5–10 h postingestion), hypotension, and hypothermia [4]. Acute FPIES is diagnosed in patients who meet the major criterion along with at least three of the minor criteria.

However, a recent cohort of patients has been identified who have milder presentations and may not meet the strict criteria listed above.

Chronic FPIES occurs in infants who have been exposed to high doses of a food allergen (e.g., feeding with cow’s milk or soy formula) and is characterized by ongoing or intermittent vomiting, watery diarrhea, and growth failure associated with the repeated ingestion of a specific trigger food. Diagnosis is confirmed when symptom resolution follows the elimination of the trigger food from the diet, and acute FPIES symptoms re-occur upon re-exposure to the food [4].

While diagnostic criteria have improved clinician awareness, growing clinical evidence has demonstrated how FPIES in newborns may present with clinical features that mimic other age-related clinical entities [4], making the diagnosis more challenging.

On the other hand, improved understanding of pathophysiology of FPIES points to the fact that specific immune pathways may underlie acute FPIES in the first weeks of life [5].

This review aims to present current insights concerning neonatal FPIES, with a special focus on clinical features, differential diagnosis, and treatment strategies, as well as to address knowledge gaps and areas for future research.

## 2. Literature Search Strategy

An extensive literature search was conducted using MEDLINE, Google Scholar, and Embase databases from inception to February 2025. The following search strings were used: “food protein induced enterocolitis” OR “FPIES” OR “allergic colitis” limited to English language, Humans, and Newborn (birth-1 month). A focused search was also performed using the terms (“food protein induced enterocolitis”) OR (“FPIES”) OR (“allergic colitis”) AND (“microbiota” OR “microbiome” OR “probiotics”), filtered by English language and Humans. Additional papers were retrieved through manual searches of references from the included documents. Guidelines, reviews, and original studies were considered, whilst studies without clear diagnostic criteria or case series with incomplete clinical data were excluded.

## 3. Epidemiology

Epidemiological data regarding FPIES are limited, due to the heterogeneity of symptoms, absence of reliable diagnostic biomarkers, and also a poor understanding of the pathogenic mechanism of the disease, leading to potential misdiagnosis and lack of recognition.

Notably, the diagnostic code for the disease (K52.21) was only established in 2015 by the International Statistical Classification of Diseases and Related Health Problems [6] and the first diagnostic guidelines were published in 2017 [3].

Data from birth cohort studies from Israel and Spain reported an incidence ranging between 0.35% [7] and 0.7% [8]. In the US, the estimated prevalence in the first year of life showed 0.5% [4], while the lowest estimated incidence of 0.015% was observed in Australia [9].

Overall, the results of these studies suggest that FPIES is not an uncommon condition as initially hypothesized, and the estimated prevalence has increased in the last decade [10].

In regard to the trigger food, cow’s milk remains the most common FPIES trigger worldwide [3].

Recently, it has been reported that the proportion of FPIES among term and preterm infants, initially suspected of having necrotizing enterocolitis, may be markedly higher than expected, among both full-term and preterm neonates who were initially diagnosed with NEC [11]. These findings point to the fact that FPIES awareness is still quite low and diagnosis remains challenging, especially in newborns.

## 4. Pathophysiology

### 4.1. Immune Mechanisms

FPIES was initially thought to be mediated by non-IgE humoral immune response and T cell-mediated cellular immunity.

The regulatory T cells (Tregs), IL-10 and TGF-β, which normally maintain immune tolerance and epithelial protection, have been found decreased in duodenal biopsies of affected infants [10,12,13].

The data regarding the involvement of humoral immunity are conflicting.

An elevated production of TNF-α, as well as Th2 cytokines like IL-6, IL-13, and IL-5, have been detected in peripheral blood mononuclear cells (PBMCs) cultured with five purified milk allergens from patients with milk-triggered FPIES, compared to healthy control in the Japanese study by Morita et al. [14].

These findings were confirmed in the study by Caubet, comparing PBMCs from patients with milk FPIES, IgE-mediated milk allergy, and controls [15]. The authors observed a similar pattern of cytokines from PBMCs in response to casein stimulation in FPIES individuals compared to controls.

However, the same cytokines were also observed in PBMCs from subjects with IgE milk allergy. This may suggest that the T cell response underlying FPIES and IgE-mediated allergy is not different.

Nevertheless, other studies did not observe any difference in the allergens’ responsive T cells in FPIES subjects compared to healthy controls [13], as well as to patients with IgE-mediated allergy [16].

It should be noted that all of the above findings were derived from in vitro studies.

However, the potential role of T cells has also been demonstrated in vivo.

In particular, the measurement of cytokines before and after FPIES challenge demonstrates an increase in IL-2, IL-17A, IL-17F, and IL-22 [17].

Th17 cells, IL-17, TNF-α, and IL-8 contribute to inflammation, increased intestinal permeability, and recruitment of neutrophils [5].

These features suggest the involvement of Th17 cells in the pathophysiology of FPIES.

Otherwise, more recent in vivo studies indicate the prevalent involvement of type 2 immune responses in neonatal FPIES. Newborns with FPIES and bloody stools showed significantly higher levels of T-helper 2 (Th2) cytokines—specifically IL-4, IL-5, and IL-13—together with elevated IL-10 when compared to patients with acute FPIES [14,18].

Notably, the Th2 predominance seems to begin prenatally in neonatal FPIES, as evidenced by significantly higher percentages and absolute counts of eosinophils in cord blood of six early onset FPIES patients compared to 30 controls (*p* = 0.0002 and *p* = 0.0001, respectively) [19]. None of the mothers suffered from cow’s milk allergy and no correlation was found between the maternal peripheral eosinophil count and what was found in the cord blood of neonates with FPIES. These findings suggest that immune priming occurs in utero, with possible increased eosinophilopoiesis; IL-5 overproduction and milk-antigen-specific Th2 cell activation would potentially develop before birth and subsequently trigger gastrointestinal inflammation upon early milk protein exposure.

As matter of fact, conspicuous eosinophilia has been observed in newborns with FPIES [20,21], experiencing both vomiting and diarrhea with bloody stools, while patients with acute FPIES did not show a rise in peripheral eosinophil count in FPIES but more frequently an eosinophilic infiltration and the deposition of eosinophil major basic protein in affected intestinal tissues [1,22].

The innate immune system also seems to play an important role, as evidenced by the presence of neutrophils, eosinophils, and lymphocytes in fecal samples and a significant increase in circulating neutrophils following FPIES reactions, accompanied by elevated serum levels of the neutrophil chemoattractant IL-8 [15,21]. According to a neonatal case-control and validation cohort study, serum IL-27 significantly discriminates necrotizing enterocolitis from neonatal FPIES with a higher performance (using a receiver operating characteristic (ROC) curve) compared to IL-6, C-reactive protein, white blood cell count, neutrophils, lymphocytes, neutrophil to lymphocyte ratio, and platelet count [23].

Though recent evidence has better elucidated the pathways underlying FPIES and the prevalent role of Th2 in neonatal FPIES, the complex interplay between innate and adaptive immunity remains far from clarification and needs to be further investigated.

### 4.2. Neurophysiological Mechanisms

Recent studies have expanded our understanding of FPIES pathophysiology, highlighting the role of neuroimmune interactions in PFIES. During symptomatic oral food challenges, Berin et al. [17] identified a distinctive inflammatory profile dominated by IL-17, IL-22, and IL-2, coupled with increased REG1A protein expression, which regulates intestinal barrier function. This IL-17-driven inflammatory signature may trigger the nervous system, leading to the characteristic symptoms of FPIES. A key connection between immune activation and clinical symptoms seems to be mediated by serotonin (5-HT) signaling. The effectiveness of ondansetron, a serotonin receptor blocker, in stopping acute FPIES episodes supports this hypothesis [24].

Additionally, T lymphocytes can produce serotonin [25], providing a direct immune-to-neural connection. Metabolomic analyses have also shown higher levels of purine pathway metabolites and 5-hydroxyindole acetate (a serotonin breakdown product) during FPIES reactions [26]. This suggests that exposure to trigger foods causes immune activation, leading to serotonin release, gastrointestinal dysmotility, and characteristic FPIES symptoms [27,28].

However, the link between innate inflammation and the activation of neural pathways that lead to vomiting is far from understood.

### 4.3. Gut Microbiota

Growing evidence is emerging that early life gut dysbiosis may play an important role in the development of an allergy [29,30] in utero and in early life. The microbiota diversity and a healthy ecosystem foster the barrier integrity and modulate the immune system towards a protective and tolerogenic milieu [31]. Distinct diet, nutritional intervention, and “biotics” supplementation have been attempted to shape the intestinal microbiome and metabolome to prevent the development of inflammatory, autoimmune, and allergic diseases [30,32].

We identified three studies investigating the fecal microbiota composition in children with FPIES [33,34,35]. In a prospective birth cohort study that enrolled 874 children followed for 3 years, 8 FPIES cases (3 related to oat, 3 to rice and 2 to cow’s milk) were identified. The 16S ribosomal RNA (rRNA) sequencing revealed lower diversity and a distinct stool microbiome profile in cases compared to 77 age-matched control children both 0–3 and 4–6 months of age. At the phylum levels, FPIES children showed higher Bacteroides and less abundance of Actinobacteria and Firmicutes. At the species level, Bacteroides fragilis, Ruminococcusbromii, Parabacteroides distasonis, and Clostridium baratii were all more abundant in children with FPIES at 0–3 months of age, whilst significantly fewer fecal Bifidobacterium spp., especially Bifidobacterium adolescentis were noted. A higher abundance of pathobionts was also identified in the stools from FPIES children, including Clostridium perfringens, Bacteroides caccae, Ruminococcusbromii, Gemmigerformicilis, and Bacteroides ovatus at 0–3 months of age and Acinetobacter lwoffii at 4–6 months of age. Children with FPIES showed less fecal short-chain fatty acid (SCFA)-producing bacteria (Bifidobacterium adolescentis, Veillonella parvula, Ruminococcusgnavus, Clostridium neonatale, Clostridium butyricum, Rothiamucilaginosa, Roseburiafaecis, and Faecalibacteriumprausnitzii). Applying a random forest machine learning model, Bifidobacterium spp., Bifidobacterium adolescentis, *Roseburia* spp., and Bacteroides fragilis differentiated children with FPIES from controls [33]. The rate of initial breastfeeding was similar between the two groups of infants (50% in FPIES vs. 60% without FPIES, *p* = 0.72) [36].

A case-control study assessed the fecal microbiota profiles of 29 children using 16S rRNA sequencing (gene hypervariable V4–V5 regions). Compared to 12 age-matched controls, the 17 children with FPIES (65% due to fish) showed a significantly higher proportion of *Lachnospiraceae* spp. and a lower proportion of *Ruminococcaceae* spp., *Lactobacillaceae* spp., and *Leuconostocaceae* spp. [34]. Among the 17 cases with FPIES, exclusive breastfeeding was reported in 2 infants, formula feeding in 1 and mixed feeding in 13 infants, compared to 5 infants exclusively breastfed and 7 with mixed feeding in the control group [34].

Recently, the gut microbiota and the level of fecal fatty acids of 12 infants with CM FPIES and of 14 matched healthy controls were investigated by 16S amplicon and shotgun sequencing and by gas chromatography. *Actinomycetota*, *Bifidobacteriaceae*, and Bifidobacterium were significantly more abundant in the fecal samples of the control group infants, whilst *Pseudomonadota* (earlier known as *Proteobacteria*), Enterobacteriaceae, Klebsiella, and *Escherichia* were significantly higher in infants with FPIES [36].

No significant differences were detected between FPIES and control infants when analyzing gender, mode of delivery, and type of feeding [36].

The authors found a positive association between bifidobacteria and the levels of interleukin (IL)-1 receptor antagonist protein (IL-1ra), the cytokine interferon gamma inducible protein-10 (IP-10), and the platelet-derived growth factor BB (PDGF-bb), which were significantly reduced in the FPIES group [36]. The concentration of fecal fatty acids, e.g., acetic, isovaleric, and isobutyric acids, were significantly higher in patients compared to controls, and that was interpreted as possibly being related to impaired colonocyte absorption induced by Proteobacteria proliferation [35].

In conclusion, current data highlighted a distinct microbiota composition in children with FPIES, detectable even before the clinical symptoms onset and characterized by a reduced diversity; less abundance of commensal Bifidobacterium, Clostridium species, and bacteria producing SCFA; and more pathobiont species compared to control groups. Overall, this microbiota signature could in turn predispose the child to barrier dysfunction, reduced regulatory T cells, increased intestinal inflammation, altered colonocyte function, and abnormal intestinal production of serotonin responsible for the symptoms of FPIES [37,38,39]. Hence, if distinct microbial features are found to be associated with FPIES, then GI microbiome manipulation by diet, probiotics, prebiotics, symbiotic, postbiotics, and/or microbiota transfer could be considered as potential modalities for the prevention and/or treatment of FPIES.

## 5. Risk Factors

Genetic predisposition appears to play a more significant role than previously recognized. A US population-based study reported 4.9% of cases had multiple siblings affected and 1.9% had an affected parent [40]. Similarly, an Australian population-based study found 7% of infants with FPIES had an affected sibling, calculating a risk of 15.4/100,000 per year without an affected sibling versus 16.4/100,000 per year with an affected sibling—suggesting a modest hereditary component [9,40]. Multiple case reports of affected monozygotic twins [9,40,41,42,43] further support a genetic contribution, highlighting the need for further studies. However, genetic factors alone do not fully explain FPIES development, as shown by reports of monochorionic twins where only one is affected [44]. Some reports have explored a possible association between Down syndrome and FPIES. In Italian and Japanese cohorts of children with trisomy 21, approximately 11% were diagnosed with FPIES to various foods—a greater than 10-fold higher rate than population estimates. The potential mechanism may relate to four genes located on human chromosome 21q22.11 that encode subunits of the IFN-α receptor, IL-10 receptor, and IFN-γ receptor. Overexpression of these genes leads to increased TNF-α and IFN-γ levels with decreased IL-10 concentration, potentially creating exaggerated inflammatory responses [45,46].

Furthermore, surgical history, such as colostomy, and postoperative cow’s milk formula nutrition might increase the risk in this population [47].

The role of sex as a risk factor is unclear. Some studies indicate that FPIES is more common among boys [48,49,50,51,52], while others do not [53,54,55,56]. Overall, it seems that there may be a weak male predominance in infants and female predominance in adults [57].

Multiple studies suggest high rates of comorbid atopic disease in FPIES patients, including IgE-mediated food allergies, atopic dermatitis, allergic rhinitis, and asthma [40,58]. A Philadelphia cohort study found statistically significant higher rates of atopic disease in those with FPIES compared to the general population, with the largest difference in IgE-mediated food allergies (24% in FPIES vs. 4% in the general population) [58].

In regard to environmental factors, the mode of delivery has long been proposed to influence the development of food allergy and atopy [59].

A higher prevalence of delivery via caesarean section was observed in infants with cow’s milk FPIES, two times higher compared to vaginal delivery [7].

This observation has been confirmed by a large cohort study of 13,019 Jewish newborns that showed a weak association between FPIES and cesarean delivery [7].

These findings raised the hypothesis that the disruption of maternal transference of microbiota to the infant may play a role in the development of FPIES, increasing the research interest in the field of microbiota [7].

Notably, the consumption of antibiotics during pregnancy may represent a further risk factor, as pointed out by a recent survey of parents or guardians of infants with and without FPIES in the first 12 months of age, which revealed higher prenatal maternal antibiotic usage in the FPIES group than controls [60].

If these results are confirmed by further prospective studies, then there is room for intervention in clinical practice.

In regard to dietary factors, no association between maternal dietary intake and development of FPIES has been observed [39].

Breastfeeding seems to play a protective role against FPIES, since FPIES is rare in exclusively breastfed infants, who prove asymptomatic until direct exposure with the causal food [61].

The protective role of breastfeeding is probably due to the presence of predigested and partially digested food antigens as well as transforming growth factor-β, IgA, and other bioactive molecules. Additionally, longer duration of breastfeeding has been found to be associated with a decreased risk in the development of severe reactions [6].

In regard to the timing of food introduction, there is no evidence that a complementary diet containing peanut and egg increases the risk of FPIES. One of the earliest pieces of evidence [62] seems to support this hypothesis, while other studies did not confirm it.

In general, feeding practice and risk of FPIES have been poorly investigated and warrant further prospective investigation [62].

## 6. Clinical Features

Recent observations showed that FPIES in neonatal age, meaning the first four weeks after birth, may not completely fulfill the diagnostic criteria of acute FPIES, referred to in the Consensus Guidelines [5]. For instance, feeding intolerance, abdominal distension with or without bloody stools, along with repetitive vomiting, seem prominent clinical features of FPIES in the first weeks of life. The most common triggers for N-FPIES are CM and soy protein [1,2]. Symptoms usually develop within the first two weeks after birth in term babies [5,11,63], whereas they present slightly later in preterm newborns [11], aligning closely with early feeding exposure. Notably, a significant negative correlation between postnatal age at onset and gestational age has been reported for premature patients [11]. Early studies focusing on premature babies showed symptom onset of FPIES after 30–32 weeks of corrected gestational age [64]. These findings have been confirmed by more recent studies [44,65]. This is likely due to the immune system maturation, which occurs at approximately 30–32 weeks of corrected gestational age. In contrast, more than 85% of NEC occurs in preterm infants with a gestational age of less than 32 weeks or a birth weight of less than 1500 g [66,67,68]. Prematurity is the most important risk factor for NEC because of immature intestinal barriers and local host defenses, such as secretory immunoglobulin A and mucosal enzymes. In terms of a clinical picture, FPIES in newborns seems to be more similar to necrotizing enterocolitis than to acute FPIES of infants and children, especially in premature babies, making the diagnosis challenging. Both are characterized by feeding intolerance, recurrent vomiting, and bloody stools [58,69] N-FPIES may even present with compromised general conditions due to dehydration with hypovolemic shock, hypoalbuminemia, metabolic acidosis, and hypothermia, representing a medical emergency in this vulnerable population [70,71]. Such severe presentation may be misdiagnosed as IgE-mediated anaphylaxis [72] or sepsis. Evidence revealed that a notable percentage of newborns initially diagnosed with NEC were later reclassified as FPIES, particularly among term infants [13]. This suggests that the prevalence of N-FPIES may be higher than previously estimated. Lastly, in 2019, Ichimura presented a case of fetal FPIES and suggested that not only the sensitization to an antigen may occur in utero, but also that early N-FPIES may show some intrauterine symptoms. In his report, the in utero patient had significant intestinal distension on ultrasonography and MRI (at 32 weeks’ gestation) and bloody stool after initial feeding [73].

## 7. Diagnosis of Neonatal FPIES

**Figure 1:**
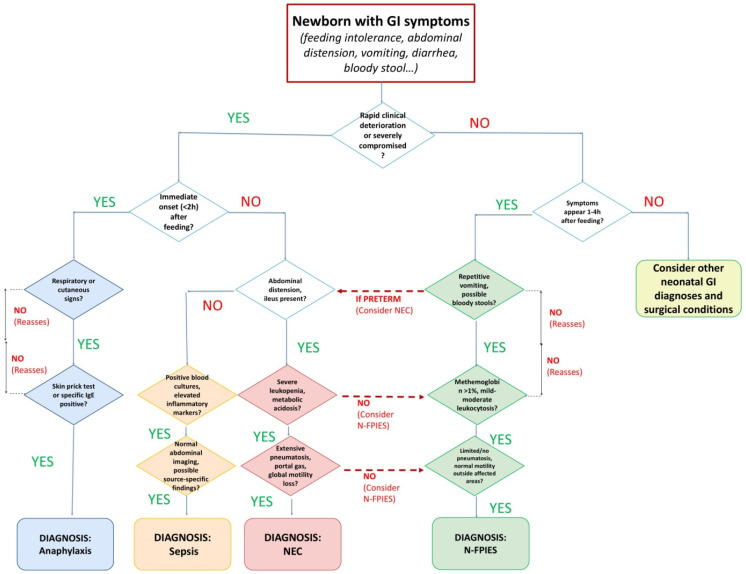
Differential diagnosis flowchart.

The diagnosis of FPIES in newborns may be particularly challenging, more so than in infants, due to the fact that the clinical picture may raise the suspicion of other conditions that need to be excluded (see Table 1 and Figure 1).

On the other hand, symptoms other than vomiting may be prominent in the neonatal period, which may lead to a delayed diagnosis of FPIES [15]. It should be highlighted that neonatal FPIES may also present with diarrhea, as a predominant feature instead of vomiting, which may also be present but not as the main symptom [63].

Diarrhea in neonatal FPIES is often severe and, in some cases, associated with visible bloody stools and poor growth. The incidence of bloody stools varies greatly when comparing data from different countries: in Western studies, 4–10% of patients with FPIES exhibited bloody stools, while Nomura and colleagues found 47% of Japanese patients exhibiting macroscopic hematochezia [74].

The diagnosis is and still remains clinical, relying on awareness and clinical expertise of clinicians.

In recent years, recognition of neonatal FPIES has been increasing in several countries, where some patients initially diagnosed with necrotizing enterocolitis (NEC) were found to possibly have FPIES instead [11,44,65].

These findings indicate that awareness of neonatal FPIES is increasing among neonatologists worldwide.

There is no specific laboratory test pathognomonic of FPIES; skin allergy tests and food-specific IgE determinations are typically negative. Several diagnostic tests have been proposed, such as detecting eosinophils in stool samples (Charcot–Leyden crystals) and the lymphocyte stimulation test (increase of casein and beta-lactoglobulin levels). However, these tests are not widely available [48].

Other laboratory findings that can be present in N-FPIES often include leukocytosis with a characteristic eosinophilic predominance, especially in preterms, along with thrombocytosis, which seems more frequent than thrombocytopenia observed in NEC [44].

It should be pointed out that peripheral eosinophilia has been described both in term and preterm newborns with FPIES [65,69], making it useful to distinguish NEC from FPIES.

In neonatal units, blood gas analysis detecting methemoglobinemia has been identified as a potential marker for severe FPIES. Decreased catalase activity during acute intestinal inflammation increases intestinal nitrites, leading to oxidative stress and methemoglobinemia [75,76]. A Japanese study established a methemoglobin cutoff level of 1% for diagnosing FPIES, with a sensitivity of 72.7% and specificity of 97.1% [75]. Makita et al. have shown that methemoglobin levels seems to be higher in patients with neonatal-onset FPIES than in those with other gastrointestinal diseases [75].

Radiographic findings also help differentiate NEC from FPIES. NEC is commonly characterized by pneumatosis intestinalis, portal venous gas, and diffuse loss of intestinal motility. Abdominal ultrasounds reveal thickened bowel walls, absent intestinal motility, and fluid collections across the abdomen [77].

In contrast, FPIES is characterized by more localized bowel involvement, with normal intestinal motility outside the affected area. While pneumatosis intestinalis and portal venous gas can also be seen in FPIES, these findings are typically less extensive and resolve more quickly compared to NEC [77]. Furthermore, patients with FPIES usually have less leukopenia and thrombocytopenia, more eosinophilia, and are less likely to have an elevated CRP [65]. Management strategies highlight the importance of accurate diagnosis. NEC requires immediate medical intervention, including bowel rest (fasting), broad-spectrum antibiotics, and, in severe cases, surgical resection of necrotic bowel tissue. Delayed or inappropriate treatment can lead to significant complications, including long-term gastrointestinal sequelae or death. In comparison, FPIES is managed by removing the offending dietary protein and substituting it with extensively hydrolyzed or amino acid-based formulas. Unlike NEC, unnecessary fasting and antibiotic use in N-FPIES can exacerbate symptoms or delay recovery [11,77].

A challenge test with the suspected allergen can confirm the diagnosis of FPIES.

However, the oral food challenge (OFC) test, given the moderate to severe nature of symptoms in the neonatal population, is often avoided in the early stages to prevent exacerbation [63].

Recent investigation demonstrated the absence of vomiting at relapsing on the reintroduction of the triggering food in FPIES in selected preterm babies, making the diagnosis questionable [18]. In these cases, performing the OFC test allowed clinicians to diagnose FPIES and thereby the required diet elimination. Thus, the need for performing the OFC test in neonatal FPIES remains an issue to be further investigated in larger studies.

## 8. Treatment of Neonatal FPIES

In N-FPIES that presents with repetitive vomiting, enteral feeding should be stopped and intravenous hydration should be started to prevent dehydration and electrolyte imbalance. For patients with severe symptoms consistent with shock (i.e., presenting lethargy, hypotension), intravenous fluid boluses followed by maintenance fluids should be administered and vital signs should be monitored closely. Rapid clinical deterioration should be considered more suspicious of NEC than FPIES, and antibiotic treatment should be promptly started. In N-FPIES, the interruption of enteral feeding usually leads to rapid improvement in general clinical conditions along with vomiting cessation. Elimination of the dietary protein trigger and supporting the infant’s nutritional needs and recovery are further key steps in N-FPIES management. The most common trigger food for neonatal FPIES is CM; in addition, there are rare cases induced by breast milk [63]. For breastfed infants, maternal dietary modification is recommended, with the elimination of the suspected protein, such as CM or soy, from the mother’s diet [63]. Prompt identification and removal of the trigger often leads to rapid gastrointestinal symptom resolution. For severe the case who does not respond to maternal diet exclusion, interruption of breast milk may be necessary, requiring a switch to extensively hydrolyzed or amino-acid based formula [78]. In cases of severe presentation, such as dehydration or metabolic acidosis from recurrent vomiting and diarrhea, supportive care is necessary, including fluid and electrolyte infusion to stabilize the patient. For neonates who are not breastfed, the primary intervention consists of transitioning the infant to an extensively hydrolyzed protein formula or an amino acid-based formula; the latter has been demonstrated to be tolerated in almost all preterm newborns [44,65], likely due to the immature digestive and immune systems of this vulnerable population. Thus, the treatment of choice as the first line intervention in premature babies with FPIES still needs to be determined (see Table 2). It should be remembered that fasting and antibiotic therapy are not required for the treatment of N-FPIES, as they can exacerbate the condition by altering the gut microbiota and delaying recovery [65].

Nutritional monitoring and gradual reintroduction of foods under medical supervision may also be an essential part of long-term management in order to avoid unnecessary treatments and growth complications, ensuring a favorable outcome [63].

## 9. Strengths and Weaknesses

This review critically summarizes the current knowledge on neonatal FPIES and highlights the involvement of neuroimmune mechanisms, intestinal microbiota, diet, and genetic and environmental factors. In addition, the various clinical presentations, differential diagnosis, diagnostic challenges, and treatment strategies are discussed to provide updated information that can help clinicians in the diagnosis and management of patients with FPIES onset in neonatal age. However, there are still few and heterogeneous studies focusing on this condition that may occur during this period of life. Furthermore, most of the available studies have small sample sizes and incomplete data at diagnosis and follow-up, which limit the conclusions and recommendations for clinical practice.

## 10. Conclusions

Neonatal food protein-induced enterocolitis presents unique clinical pictures and may not necessarily fulfill the full diagnostic criteria for acute or chronic FPIES described in the international consensus guidelines.

Due to the similarity in symptoms, neonatal FPIES may sometimes be misdiagnosed as necrotizing enterocolitis, especially in preterm newborns. In addition, chronic FPIES may develop in the first few weeks after birth, with severe diarrhea and poor growth as predominant symptoms. Although the knowledge of acute and chronic FPIES has improved over the years, clinician awareness of neonatal FPIES is still quite low, possibly leading to misdiagnosis.

Hence, refining diagnostic criteria for neonatal FPIES represents a clinical priority to help physicians properly diagnose and treat this challenging condition (see Table 3).

Furthermore, larger clinical trials are needed in order to optimize treatment practices in term and preterm newborns with FPIES.

## Figures and Tables

**Table 1:** Differential diagnosis of N-FPIES.

Condition	Key Features
Necrotizing Enterocolitis (NEC)	Onset in the first weeks after birth more frequently in preterm infants with EG <32 wks; unstable temperature or fever; lethargy, vomiting, abdominal distension or bloating, bloody stools; pneumatosis intestinalis on imaging; absence of peripheral eosinophilia
Sepsis	Positive cultures, severe clinical presentation, unstable temperature or fever, poor appetite, respiratory distress or diarrhea or reduced bowel movements, jaundice; evidence of systemic inflammation, hypoglyceamia; improvement with antibiotics; not food-specific
IgE-Mediated Allergy/Anaphylaxis	Immediate onset after food exposure; associated respiratory and/or cutaneous manifestations and/or vomiting; hypotension and eventually shock if anaphylaxis; positive food-specific IgE or skin prick tests
Surgical conditions	Delayed meconium passage (Hirschsprung), abdominal distension; bilious or fecaloid vomiting; signs of intestinal obstruction; possible bloody diarrhea
Congenital Metabolic Disorders	Vomiting, progressive neurologic deterioration, hypotonia, lethargy, liver dysfunction, hypoglycemia, acidosis, poor growth
Immune deficiencies	Recurrent or severe infections; failure to thrive; frequent cutaneous manifestations
Neonatal Onset Inflammatory Bowel Disease (N-IBD)	Early onset severe diarrhea, often bloody and/or mucus-containing diarrhea, frequent emesis, perianal skin tags or fistulas, systemic symptoms and/or extraintestinal symptoms, failure to thrive, absence of infectious etiology, possible genetic mutations, possible improvement with elemental amino-based formula

**Table 2:** Dietary management of neonatal FPIES.

Formula Selection	Extensively Hydrolyzed Formula (eHF)	First-line choice for most cases.
	Amino Acid-Based Formula (AAF)	Used if no improvement is observed with eHF or poor growth within 2 weeks; consider as first choice in preterm infants
Breastfeeding	Continue Breastfeeding if Tolerated	If symptoms persist, maternal elimination diet should be considered
	Eliminate Trigger Food (dairy and soy) for 2–4 weeks.	Monitor infant’s symptoms to assess improvement
	Temporary Switch to Hypoallergenic Formula (eHF)	Consider this option only for severe cases who do not respond to maternal diet exclusion
Nutritional Counseling	Individualized Dietary Plan	Ensure optimal growth and nutrition, avoiding unnecessary dietary restrictions
	Monitor Growth and Development	Regular follow-up to assess weight gain

**Table 3:** Knowledge gaps and future research perspectives.

Knowledge Gaps	Future Research Perspectives
The prevalence of neonatal FPIES remains unclear	Further studies involving neonatologists are needed in order to clarify the prevalence of neonatal FPIES in different countries
Current poor knowledge of the risk factors for N-FPIES	Deeper knowledge of modifiable risk factors, in particular the timing of milk introduction, may help to guide dietary management and feeding strategies
Lack of diagnostic biomarkers	Improving pathophysiology knowledge to possibly help identify diagnostic and prognostic biomarkers
Reluctance and limited availability for OFC practice in newborns	Need for standardized oral food challenge protocols for diagnosing FPIES in newborns
The preferred first-line treatment for preterm newborns is not well known	Randomized studies comparing extensively hydrolyzed formula (eHF) with amino acid formula (aa) should be conducted in order to determine the first-line treatment of choice
